# Epidemiology and outcomes of previously undiagnosed diabetes in older women with breast cancer: an observational cohort study based on SEER-Medicare

**DOI:** 10.1186/1471-2407-12-613

**Published:** 2012-12-22

**Authors:** Robert I Griffiths, Mark D Danese, Michelle L Gleeson, José M Valderas

**Affiliations:** 1 Department of Epidemiology, Outcomes Insights, Inc, 340 N. Westlake Blvd, Suite 200, Westlake Village, CA 91362, USA; 2 Division of General Internal Medicine, Johns Hopkins University School of Medicine, Baltimore, MD, USA; 3 Department of Primary Care Health Sciences, University of Oxford, Oxford, UK

**Keywords:** Breast cancer, Diabetes, Previously undiagnosed, Risk factors, Stage, Mortality, Survival

## Abstract

**Background:**

In breast cancer, diabetes diagnosed prior to cancer (previously diagnosed) is associated with advanced cancer stage and increased mortality. However, in the general population, 40% of diabetes is undiagnosed until glucose testing, and evidence suggests one consequence of increased evaluation and management around breast cancer diagnosis is the increased detection of previously undiagnosed diabetes. Biological factors – for instance, higher insulin levels due to untreated disease - and others underlying the association between previously diagnosed diabetes and breast cancer could differ in those whose diabetes remains undiagnosed until cancer. Our objectives were to identify factors associated with previously undiagnosed diabetes in breast cancer, and to examine associations between previously undiagnosed diabetes and cancer stage, treatment patterns, and mortality.

**Methods:**

Using Surveillance, Epidemiology, and End Results-Medicare, we identified women diagnosed with breast cancer *and diabetes* between 01/2001 and 12/2005. Diabetes was classified as previously diagnosed if it was identified within Medicare claims between 24 and 4 months before cancer diagnosis, and previously undiagnosed if it was identified from 3 months before to ≤ 3 months after cancer. Patients were followed until 12/2007 or death, whichever came first. Multivariate analyses were performed to examine risk factors for previously undiagnosed diabetes and associations between undiagnosed (compared to previously diagnosed) diabetes, cancer stage, treatment, and mortality.

**Results:**

Of 2,418 patients, 634 (26%) had previously undiagnosed diabetes; the remainder had previously diagnosed diabetes. The mean age was 77.8 years, and 49.4% were diagnosed with *in situ* or stage I disease. Age > 80 years (40% of the cohort) and limited health system contact (primary care physician and/or preventive services) prior to cancer were associated with higher adjusted odds of previously undiagnosed diabetes. Previously undiagnosed diabetes was associated with higher adjusted odds of advanced stage (III/IV) cancer (Odds Ratio = 1.37: 95% Confidence Interval (CI) 1.05 – 1.80; P = 0.02), and a higher adjusted mortality rate due to causes other than cancer (Hazard Ratio = 1.29; 95% CI 1.02 – 1.63; P = 0.03).

**Conclusions:**

In breast cancer, previously undiagnosed diabetes is associated with advanced stage cancer and increased mortality. Identifying biological factors would require further investigation.

## Background

Epidemiologic evidence suggests pre-existing diabetes is associated with increased risk of breast cancer [1], advanced cancer stage at diagnosis [2-5], altered treatment regimens [2,6-8], chemotherapy toxicity [6], breast cancer mortality in the general population [1,9,10], and overall mortality in those diagnosed with breast cancer [2-8,11]. Evidence supporting the association between pre-existing diabetes and overall mortality in breast cancer is extensive. Recently, Peairs and colleagues [2] conducted a systematic review and meta-analysis in which they combined results from 6 studies [6-8,12-14], and found diabetes was associated with a 49% increased risk of death due to all causes. Studies on the association between diabetes and cancer mortality in those diagnosed with breast cancer have produced inconsistent findings [2,6,11,15]. One based on the National Cancer Institute’s (NCI) Surveillance, Epidemiology, and End Results (SEER) – Medicare database showed diabetes was associated with a 10% increase in breast cancer deaths [11]; in another, only those who received adjuvant chemotherapy were at significantly increased risk [6]; while in a third [15] there was no association between pre-existing diabetes and cancer mortality.

Biological links between diabetes and breast cancer risk and outcomes include hyperinsulinemia, hyperglycemia, and chronic inflammation [16-18]. Hyperinsulinemia related to underlying insulin resistance stimulates tumor growth, working directly on epithelial cells or indirectly by activating insulin-like growth factor pathways or altering endogenous sex hormones [2]. Several other factors may link diabetes to breast cancer outcomes: presentation with later-stage cancer due to suboptimal breast cancer screening practices [19,20] or other health-seeking behavior [21-23]; interactions in the management of the two conditions, including less aggressive breast cancer treatment due to diabetes-related comorbidity [6,7]; poorer response to treatment; and, possibly, that the diagnosis of breast cancer may distract both the patient and the health care team from the appropriate management of glycemia [24].

Thus far, most epidemiology studies of diabetes and breast cancer outcomes have classified patients as having diabetes if it was diagnosed prior to cancer, including several studies based on SEER-Medicare [6,11,15] that identified diabetes from Medicare claims [25,26] during 12 months prior to cancer. However, in the general adult population, approximately 40% of diabetes remains undiagnosed until glucose testing [27], and there is also evidence many diabetes cases may remain undiagnosed until breast cancer [28]. Recently, we conducted a study in SEER-Medicare to examine the impact of breast cancer diagnosis on the detection of other previously undiagnosed conditions, including diabetes [28]. The prevalence of pre-existing diabetes in the cancer patients was 14.3%, and it was similar in a cohort of matched controls (12.8%). However, the incidence of undiagnosed diabetes was 35.0/1,000 compared to only 13.5/1,000 after a matched sham date in the controls, suggesting that one consequence of increased evaluation and management around breast cancer diagnosis is the detection of previously undiagnosed diabetes. Furthermore, Erickson and colleagues [5] found that of breast cancer patients with hemoglobin A1C (HbA1C) ≥ 7% (n=91), only 40.7% indicated they had diabetes on a baseline self-report questionnaire; only 10% of those with HbA1C of ≥ 6.5% - a current criterion for the diagnosis of diabetes [29] - self-reported diabetes. One implication of these findings is that studies on the outcomes of pre-existing diabetes in breast cancer may contain in their control groups many patients with undiagnosed diabetes.

Biological and other links between diabetes and outcomes in breast cancer may differ between those with previously undiagnosed compared to previously diagnosed diabetes. Hyperinsulinemia could be exacerbated in those with previously undiagnosed, and presumably untreated, diabetes, leading to more aggressive tumor growth. Also, there is evidence that some diabetes treatments influence cancer risk and prognosis. Metformin, the most commonly used therapy for type II diabetes, is often prescribed as initial mono- or combination therapy [17]. Preclinical data show an *in vitro* effect of metformin in breast cancer cells [2,30], and in an observational study in humans, metformin was associated with a higher pathologic complete response among early-stage breast cancer patients receiving neoadjuvant therapy [31]. In contrast to the protective effect of metformin, exogenous insulin use could promote tumor growth resulting in more advanced stage cancer at diagnosis among those with previously diagnosed and treated diabetes.

Data directly supporting the hypothesis that breast cancer outcomes differ between those with pre-existing and previously undiagnosed diabetes are scarce. Findings from a study based on the second National Health and Nutrition Examination Survey (NHANES) do suggest cancer mortality in patients with previously undiagnosed diabetes may be higher than in previously diagnosed diabetes, where undiagnosed diabetes was detected through oral glucose tolerance testing [32]. However, this study was conducted in the general population, the two diabetes groups were not compared directly, and breast cancer was not assessed separately. Data on the incidence and risk factors for previously undiagnosed diabetes in cancer also are scarce. In a SEER-Medicare study, we reported that detection of many chronic conditions, including diabetes, increases around the time of breast cancer diagnosis [28], but a detailed examination of risk factors for previously undiagnosed compared to previously diagnosed conditions was beyond the scope of that study.

The objectives of the present study were (A) to identify demographic, socioeconomic, and clinical factors associated with previously undiagnosed, compared to previously diagnosed, diabetes in a cohort of breast cancer patients, all of whom had diabetes, and (B) to examine associations between previously undiagnosed, compared to previously diagnosed, diabetes and stage at breast cancer diagnosis, treatment patterns, and mortality.

## Methods

### Data source

The source of data for this study was SEER-Medicare [33]. Presently, SEER contains cancer incidence and survival data from 17 population-based cancer registries throughout the United States covering approximately 28% of the population [34]. In SEER-Medicare, cancer registry data are linked to Medicare enrollment and claims data, which are available for 93% of those aged ≥ 65 years in the SEER registry [35].

### Inclusion and exclusion criteria

Patients were included if they were diagnosed with breast cancer between January 1, 2001, and December 31, 2005, breast was the first and only type of cancer at the time they were diagnosed, they met the minimum age requirement for Medicare eligibility (65 years), they had at least 24 months of Medicare Part A (hospital) and Part B (outpatient) fee-for-service coverage prior to the diagnosis of cancer, *and they were diagnosed with diabetes between 24 months before and 3 months after cancer diagnosis*. We restricted the cohort to those with Part A and B coverage because the vast majority of inpatient and outpatient services for these patients are captured within the SEER-Medicare database. Patients were excluded for the following reasons: male breast cancer; cancer diagnosis made by death certificate or autopsy; death within the first month following diagnosis; or qualification for Medicare based on disability alone. Requiring all patients to be at least 65 years old at diagnosis and to have at least 24 months of Medicare coverage prior to cancer diagnosis meant that the minimum age at cancer diagnosis in the study was 67 years.

### Observation period

Patients were followed from 24 months before cancer until the end of the claims period (December 31, 2007) or death or the occurrence of a second primary cancer, whichever came first. Since SEER reports only the month of diagnosis, the first day of that month was assigned as the date of diagnosis.

### Diabetes

Diabetes was defined as the presence of one or more of the following International Classification of Diseases, 9^th^ Revision, Clinical Modification, (ICD-9-CM) diagnosis codes in any position in any Medicare claim: 250.xx for diabetes and complications; 357.2x for polyneuropathy in diabetes; 362.0x for diabetic retinopathy; and 366.41 for diabetic cataract [25]. This validated algorithm has been used in other studies of pre-existing diabetes in breast cancer [6], and has a sensitivity of 74.4% and specificity of 97.5% using a 2-year look-back period [25]. Laboratory claims were excluded to reduce the likelihood of misclassifying as diabetes cases those patients only undergoing diagnostic evaluation for suspected diabetes. We did not include diabetes medications in the definition since Medicare did not begin covering oral medications without an intravenous equivalent until January, 2006.

Patients were classified as having previously diagnosed diabetes if the first diabetes claim qualifying them for inclusion in the study was between 24 and 4 months (inclusive) prior to cancer diagnosis. They were classified as having previously undiagnosed diabetes if their first diabetes claim was between three months before and three months (inclusive) after cancer diagnosis, or the beginning of radiation or chemotherapy, or death, whichever came first.

### Patient characteristics

Patients were described according to their demographic, clinical, and socioeconomic characteristics. Stage at cancer diagnosis was based on the SEER-Modified American Joint Committee on Cancer (AJCC) stage variable [36]. Medicare claims were used to calculate an NCI Comorbidity Index score for each patient [26,37-42]. The two conditions pertaining to diabetes were removed from the NCI Comorbidity Index to reduce correlation with previously diagnosed diabetes. Medicare claims also were used to identify several indicators of poor performance status [43], a claims-based surrogate for Eastern Cooperative Oncology Group Performance Status, including the use of oxygen and related respiratory therapy supplies, wheelchair and supplies, home health agency use, and skilled nursing facility use.

Poor prior health system contact is associated with advanced cancer stage at diagnosis [21-23], an important prognostic factor for cancer outcomes. To account for this in our analyses, we constructed two measures of prior health system contact 24 to 4 months before cancer based on this literature [21,22]. First, we constructed a physician contact index that classified patients according to the types of ambulatory care visits they received [21]. We searched the Medicare physician/supplier claims file for physician outpatient visits, and classified each visit as primary care physician (general practitioner, family practitioner, general internist, geriatrician, obstetrician/gynecologist), medical specialist, or other specialist. Other specialists included general surgeons, ophthalmologists, orthopedic surgeons, and other surgical specialists [21]. The presence of one or more claims for each type of physician visit was coded as “1” for that type. Since only one of the two Medicare outpatient services files (physician/supplier and “outpatient”) contains information on physician specialty, the absence of a primary care or specialist visit in the physician/supplier file should not be interpreted as absence of any outpatient health system contact.

Second, we constructed an index of preventive services based on one developed by Gornick et al. [22], which includes mammography, screening for colorectal cancer, Papanicolaou test, screening for glaucoma, influenza immunization, and pneumonia immunization. The presence of one or more claims for each type of service was coded as “1” for that service, and individual scores were combined in an index consisting of 0, 1, or ≥ 2. Socioeconomic information at the patient level is not available through SEER-Medicare. Instead, the dataset contains information from the 2000 Census, reported at the tract level in which the patient lives.

### Outcomes variables

We examined risk factors for previously undiagnosed compared to previously diagnosed diabetes, and assessed associations between previously undiagnosed diabetes and advanced stage (III or IV) compared to earlier stage (*in situ*, I, or II) cancer at diagnosis, time to initial chemotherapy or radiation, and mortality. We searched Medicare claims from the date of cancer diagnosis through the end of the observation period to identify ICD-9-CM and Healthcare Common Procedure Coding System codes indicating treatment with chemotherapy or radiation [44,45]. The date of the first such claim was used to indicate the beginning of that treatment.

The date of death was assigned using the Medicare date, if available, even in cases where the SEER date also was available. The Medicare date was preferred because it was more current than the SEER date [46]. Where the Medicare date was missing but the SEER date was available, the SEER date was used. All other patients were assumed to be alive at the end of the observation period (December 31, 2007) based on the fact that they were required to have Medicare Part A and Part B coverage for the entire claims period. The cause of death was classified as cancer or other-cause, using the "CODKM" variable in the SEER Patient Entitlement and Diagnosis Summary File through 2007. Cancer mortality included all deaths due to cancer (CODKM = 001-130), and not just due to breast cancer (CODKM = 046). Other-cause mortality included all other identified causes of death; e.g., CODKM = 154 “Diseases of Heart”, CODKM = 148 “Diabetes Mellitus”. However, it excluded missing or unspecified cause of death. These patients were censored at the time of death in both the cancer and non-cancer mortality analysis, but considered “events” in the analysis of all-cause mortality. Cancer and other-cause mortality were examined separately since the incremental impact of previously undiagnosed diabetes could differ between these two.

### Analyses

**Figure 1:**
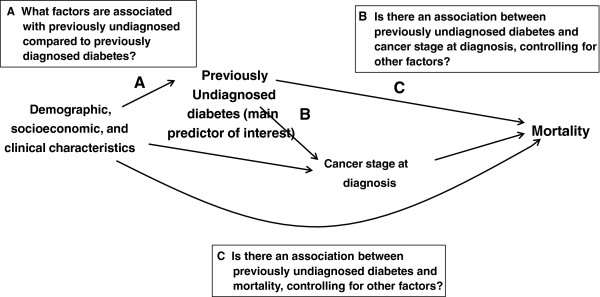
**Causal pathway diagram.** Prior to finalizing the inclusion/exclusion criteria and hypotheses for this study, a causal diagram was developed to visually encode *a priori* assumptions about the relation between exposure (previously undiagnosed versus previously diagnosed diabetes), outcomes, and covariates, taking into account the strengths and limitations of the Surveillance, Epidemiology, and End Results (SEER) - Medicare database. The diagram depicts *directed paths* (a head-to-tail sequence of arrows, or a “one-way street”) between previously undiagnosed diabetes and both cancer stage at diagnosis and mortality. One of the directed paths between previously undiagnosed diabetes and mortality contains cancer stage as an intermediate variable. In other words, the impact of previously undiagnosed diabetes on mortality is partially explained by its intermediate impact on cancer stage. The other directed path contains no intermediate variables. In addition, the diagram depicts *undirected paths* (paths in which the arrows are not all head-to-tail) between previously undiagnosed diabetes and both cancer stage at diagnosis and mortality, which “flow through” patient demographic, socioeconomic, and clinical characteristics. In this instance, the *undirected paths* between previously undiagnosed diabetes, cancer stage, and mortality are *biasing paths* (and the variables on those paths are potential confounders) for the association between exposure and outcomes *because they do not represent effects of previously undiagnosed diabetes on the outcomes*, *yet can contribute to (confound) the association between previously undiagnosed diabetes and outcomes*. These should be “blocked” either by study design, including patient selection, or by adjustment in the analyses, to maximize the likelihood that the observed residual associations between exposure and outcomes are unbiased.

We described the demographic, socioeconomic, and clinical characteristics of the cohort, both overall and stratified by previously diagnosed versus previously undiagnosed diabetes. Multivariate analysis was used to evaluate *a priori* hypotheses about factors associated with previously undiagnosed diabetes, and the relationships between previously undiagnosed diabetes and outcomes as specified in a causal pathway diagram (Figure 1) [47]. Figure 1 shows that we hypothesized a directed path (A) from a vector of demographic, socioeconomic, and clinical characteristics to previously undiagnosed diabetes. However, since there are also directed paths from *both* previously undiagnosed diabetes *and* the vector of demographic, socioeconomic, and clinical characteristics to cancer stage at diagnosis, cancer stage is a collider variable [47-49]. Conditioning on a collider can open a biasing pathway between two variables, in this case between the vector of patient characteristics and previously undiagnosed diabetes, making it appear that there is an association when in fact none exists. Therefore, in the multivariate analyses of factors associated with previously undiagnosed diabetes, we excluded cancer stage from the vector of independent variables in the models.

Figure 1 shows that there is a directed path and an undirected path (through demographic, socioeconomic, and clinical characteristics) from previously undiagnosed diabetes to cancer stage (*in situ*/I/II versus III/IV) at diagnosis. Therefore, adding confounders to a logistic regression model of previously undiagnosed diabetes and advanced stage cancer should attenuate (by blocking the undirected path) but not completely eliminate -- the directed path should remain open -- the association between previously undiagnosed diabetes and advanced stage. Since there is strong evidence linking higher levels of prior health system contact to early stage cancer diagnosis, we reasoned that these covariates could be strong confounders in the association between previously undiagnosed diabetes and cancer stage. Therefore, we estimated two logistic regression analyses to evaluate the effect of adding the measures of prior health system contact on the association between previously undiagnosed diabetes and cancer stage. Both analyses included age, race/ethnicity, year of cancer diagnosis, NCI Comorbidity Index, performance status, education, poverty, and geographic area as covariates.

Figure 1 also shows that there are two directed paths and two undirected paths between previously undiagnosed diabetes and mortality. In addition, one of the directed paths includes cancer stage at diagnosis as an intermediate variable. Therefore, the causal diagram suggests that adding measures of prior health system contact to a model that includes other covariates should attenuate, but not eliminate, the observed (and biased) association between previously undiagnosed diabetes and mortality. In addition, adding cancer stage as a covariate should block the directed path in which cancer stage is an intermediate variable, further attenuating the observed association between previously undiagnosed diabetes and mortality. However, it is important to note that blocking this directed path can be construed as over-adjustment, since the directed path is not a biasing path.

To explore these associations, we ran four sets of three multivariate survival analyses. Each set included all-cause, cancer, and other-cause mortality as independent variables, and age, race/ethnicity, year of cancer diagnosis, NCI Comorbidity Index, performance status, estrogen and progesterone receptor (ER PR) status, histology, education, poverty, and geographic area as covariates. We then added both measures of prior health system contact and cancer stage, separately and together, to the base set of covariates in order to examine their impact on the coefficient for previously undiagnosed diabetes. The base-case model included both measures of prior health system contact, but not cancer stage. During the exploratory phase of our study, we did sequential analyses in which we introduced first one and then the second measure of prior health system contact into our models. We found that while the effect of the first introduced was attenuated by the second, in almost all instances both remained statistically and clinically significant. Therefore, both were retained in the models that included prior health system contact.

## Results

The final cohort included 2,418 breast cancer patients with diabetes, of whom 1,784 (73.8%) had previously diagnosed and the remaining 634 (26.2%) had previously undiagnosed diabetes. Overall, the mean age was 77.8 years, 40.1% were age > 80 years, 49.4% were diagnosed with *in situ* or stage I disease, and 49.3% were both ER and PR positive (Table 1).

**Table 1:** Patient characteristics

			**Diabetes status at cancer diagnosis**				
	**Overall (N = 2,418)**		**Previously diagnosed (n = 1,784)**		**Previously undiagnosed (n = 634)**		**P-value**
	**N**	**% (SD)**	**n**	**% (SD)**	**n**	**% (SD)**	
**Age at cancer diagnosis (years)**							
**66-70**	326	13.5%	234	13.1%	92	14.5%	0.77
**71-75**	529	21.9%	397	22.3%	132	20.8%	
**76-80**	594	24.6%	437	24.5%	157	24.8%	
**>80**	969	40.1%	716	40.1%	253	39.9%	
**Mean & (SD) age**	77.8	6.9	77.8	7.0	77.7	6.9	
**Race/ethnicity**							
**White**	2,156	89.2%	1,584	88.8%	572	90.2%	0.47
**Black**	132	5.5%	103	5.8%	29	4.6%	
**Hispanic**	89	3.7%	64	3.6%	25	3.9%	
**Other**	41	1.7%	33	1.8%	8	1.3%	
**Year of diagnosis**							
**2001**	464	19.2%	322	18.0%	142	22.4%	0.05
**2002**	482	19.9%	347	19.5%	135	21.3%	
**2003**	466	19.3%	347	19.5%	119	18.8%	
**2004**	468	19.4%	363	20.3%	105	16.6%	
**2005**	538	22.2%	405	22.7%	133	21.0%	
**Stage at diagnosis**							
***In situ***	274	11.3%	217	12.2%	57	9.0%	<0.0001
**I**	920	38.0%	705	39.5%	215	33.9%	
**II**	786	32.5%	579	32.5%	207	32.6%	
**III**	239	9.9%	175	9.8%	64	10.1%	
**IV**	199	8.2%	108	6.1%	91	14.4%	
**Estrogen (ER) and progesterone (PR) receptor status**							
**ER and PR positive**	1,193	49.3%	881	49.4%	312	49.2%	
**ER or PR positive**	327	13.5%	238	13.3%	89	14.0%	0.89
**ER and PR negative**	324	13.4%	244	13.7%	80	12.6%	
**Unknown/missing**	574	23.7%	421	23.6%	153	24.1%	
**Histologic grade**							
**1**	447	18.5%	336	18.8%	111	17.5%	0.61
**2**	872	36.1%	652	36.5%	220	34.7%	
**3**	696	28.8%	502	28.1%	194	30.6%	
**4**	71	2.9%	49	2.7%	22	3.5%	
**Unknown/missing**	332	13.7%	245	13.7%	87	13.7%	
**NCI Comorbidity Index**							
**0**	1,051	43.5%	627	35.1%	410	64.7%	<0.0001
**1**	699	28.9%	560	31.4%	145	22.9%	
**≥ 2**	668	27.6%	597	33.5%	79	12.5%	
**Indicators of poor performance**							
**0**	1,169	48.3%	692	38.8%	477	75.2%	<0.0001
**≥ 1**	1,249	51.7%	1,092	61.2%	157	24.8%	
**Types of physician visits**							
**Primary care and medical specialist**	1,621	67.0%	1,277	71.6%	344	54.3%	<0.0001
**Primary care, no medical specialist**	381	15.8%	254	14.2%	127	20.0%	
**Medical specialist, no primary care**	226	9.3%	154	8.6%	72	11.4%	
**Other specialist only**	57	2.4%	34	1.9%	23	3.6%	
**None**	133	5.5%	65	3.6%	68	10.7%	
**Preventive services**							
**0**	236	9.8%	123	6.9%	113	17.8%	<0.0001
**1**	403	16.7%	294	16.5%	109	17.2%	
**≥ 2**	1,779	73.6%	1,367	76.6%	412	65.0%	
**Percent in census tract with some college**							
**<25%**	883	36.5%	656	36.8%	227	35.8%	0.66
**≥25%**	1,535	63.5%	1,128	63.2%	407	64.2%	
**Percent in census tract living in poverty**							
**<5%**	686	28.4%	509	28.5%	177	27.9%	0.29
**5-7%**	343	14.2%	255	14.3%	88	13.9%	
**8-12%**	542	22.4%	383	21.5%	159	25.1%	
**>12%**	847	35.0%	637	35.7%	210	33.1%	
**Type of geographic area**							
**Large metropolitan**	1,298	53.7%	952	53.4%	346	54.6%	0.74
**Metropolitan**	696	28.8%	510	28.6%	186	29.3%	
**Urban**	165	6.8%	125	7.0%	40	6.3%	
**Less urban/rural**	259	10.7%	197	11.0%	62	9.8%	

*SD*: Standard deviation. The NCI Comorbidity Index is based on claims 24 – 4 months before cancer, and excludes two diabetes-related conditions.

The multivariate analysis of factors associated with previously undiagnosed (compared to previously diagnosed) diabetes (Table 2) showed that later year of cancer diagnosis, higher NCI Comorbidity Index, ≥ 1 indicator of poor performance, at least one visit to a primary care physician or medical specialist, receiving ≥ 2 preventive services, and living in a less urban/rural area all were associated with significantly lower odds of previously undiagnosed diabetes. The results also suggest age > 80 years was associated with higher odds of previously undiagnosed diabetes; however, the odds ratio (OR) for this covariate narrowly failed to reach the commonly accepted threshold for statistical significance (P < 0.05).

**Table 2:** Multivariate analysis of factors associated with previously undiagnosed diabetes compared to previously diagnosed diabetes

	**Measures of prior health system contact not included**				**Measures of prior health system contact included**			
	**OR**	**95% CI**		**P-Value**	**OR**	**95% CI**		**P-Value**
		**Lower**	**Upper**			**Lower**	**Upper**	
**Age at cancer diagnosis (years)**								
**66-70**	**Reference**							
**71-75**	0.89	0.63	1.24	0.49	0.95	0.67	1.34	0.75
**76-80**	1.12	0.80	1.55	0.52	1.21	0.86	1.70	0.28
**>80**	1.35	0.99	1.84	0.06	1.35	0.98	1.86	0.07
**Race/ethnicity**								
**White**	**Reference**							
**Black**	0.89	0.55	1.43	0.62	0.75	0.46	1.24	0.26
**Hispanic**	1.05	0.62	1.78	0.87	1.02	0.59	1.75	0.95
**Other**	0.72	0.31	1.67	0.44	0.64	0.27	1.56	0.33
**Year of diagnosis**								
**2001**	**Reference**							
**2002**	0.76	0.56	1.03	0.08	0.75	0.55	1.03	0.07
**2003**	0.69	0.51	0.95	0.02	0.71	0.52	0.98	0.03
**2004**	0.61	0.44	0.84	<0.01	0.61	0.44	0.85	<0.01
**2005**	0.69	0.51	0.94	0.02	0.71	0.52	0.97	0.03
**NCI Comorbidity Index**								
**0**	**Reference**							
**1**	0.44	0.35	0.56	<0.0001	0.48	0.38	0.61	<0.0001
**≥ 2**	0.29	0.22	0.40	<0.0001	0.32	0.24	0.44	<0.0001
**Indicators of poor performance**								
**0**	**Reference**							
**≥ 1**	0.27	0.22	0.34	<0.0001	0.27	0.21	0.33	<0.0001
**Types of physician visits**								
**None**	**Not Applicable**				**Reference**			
**Primary care and medical specialist**					0.33	0.21	0.51	<0.0001
**Primary care, no medical specialist**					0.52	0.32	0.83	0.01
**Medical specialist, no primary care**					0.50	0.30	0.85	0.01
**Other specialist only**					0.67	0.32	1.37	0.27
**Preventive services**								
**0**	**Not Applicable**				**Reference**			
**1**					0.70	0.47	1.04	0.07
**≥ 2**					0.58	0.41	0.82	<0.01
**Percent in census tract with some college**								
**<25%**	**Reference**							
**≥25%**	0.97	0.79	1.20	0.79	0.95	0.76	1.18	0.62
**Percent in census tract living in poverty**								
**<5%**	**Reference**							
**5-7%**	0.99	0.72	1.37	0.96	0.98	0.70	1.36	0.88
**8-12%**	1.24	0.94	1.64	0.13	1.21	0.91	1.62	0.18
**>12%**	1.17	0.88	1.55	0.27	1.04	0.78	1.39	0.78
**Type of geographic area**								
**Large metropolitan**	**Reference**							
**Metropolitan**	0.82	0.65	1.04	0.10	0.85	0.67	1.08	0.18
**Urban**	0.74	0.49	1.12	0.16	0.78	0.50	1.20	0.25
**Less urban/rural**	0.72	0.50	1.03	0.07	0.69	0.48	1.00	0.05

*OR*: Odds ratio. *CI*: Confidence interval. Not Applicable: Covariates not included in that model.

### Cancer stage at diagnosis

Overall, 18.1% of all patients (n=438) were diagnosed with stage III/IV breast cancer: 15.9% (n=283) of those with previously diagnosed diabetes, and 24.4% (n=155) with previously undiagnosed diabetes (p < 0.0001 for unadjusted difference in distribution across all 5 cancer stages [Bivariate results shown in Table 1]). In a multivariate analysis (reported in the text below) that excluded measures of prior health system contact, the odds of being diagnosed with stage III/IV disease were 76% higher (OR=1.76; 95% Confidence Interval [CI] 1.37 – 2.28; p < 0.0001) for patients with previously undiagnosed diabetes (compared to previously diagnosed diabetes). When measures of prior health system contact were introduced, the OR for previously undiagnosed diabetes decreased to 1.37, but remained statistically significant (95% CI 1.05 – 1.80; p = 0.02). Both measures of prior health system contact (types of physician contact and preventive services) were statistically significant in the latter model, showing less/poor quality prior health system contact was associated with significantly increased odds of advanced stage at diagnosis. Those living in a census tract with > 12% poverty were more likely to be diagnosed with advanced stage disease, although this effect was attenuated slightly by the introduction of the aforementioned measures of prior health system contact.

### Initial treatment

Overall, 479/2,418 (19.8%) received chemotherapy: 18.6% of those with previously diagnosed diabetes and 23.3% of those with previously undiagnosed diabetes. In addition, 662/2,418 (27.4%) received radiation: 28.1% of those with previously diagnosed diabetes and 25.2% of those with previously undiagnosed diabetes. In multivariate analysis of time to initial treatment, there was no difference in time to initial chemotherapy (Hazard Ratio [HR] = 1.08; 95% CI 0.87 – 1.34; P = 0.50), radiation (HR = 0.86; 95% CI 0.71 – 1.04; P = 0.12), or either chemotherapy or radiation, whichever came first (HR = 0.89; 95% CI 0.76 – 1.04; P = 0.14), between those with previously undiagnosed and those with previously diagnosed diabetes, adjusting for all patient factors reported in Table 1. Age > 80 years at diagnosis, *in situ* or stage I disease, and being both ER and ER positive were associated with lower rates of chemotherapy and radiation. In contrast, stage III or IV (compared to stage II, the reference category) disease was associated with higher rates (HRs not shown).

### Mortality

**Figure 2:**
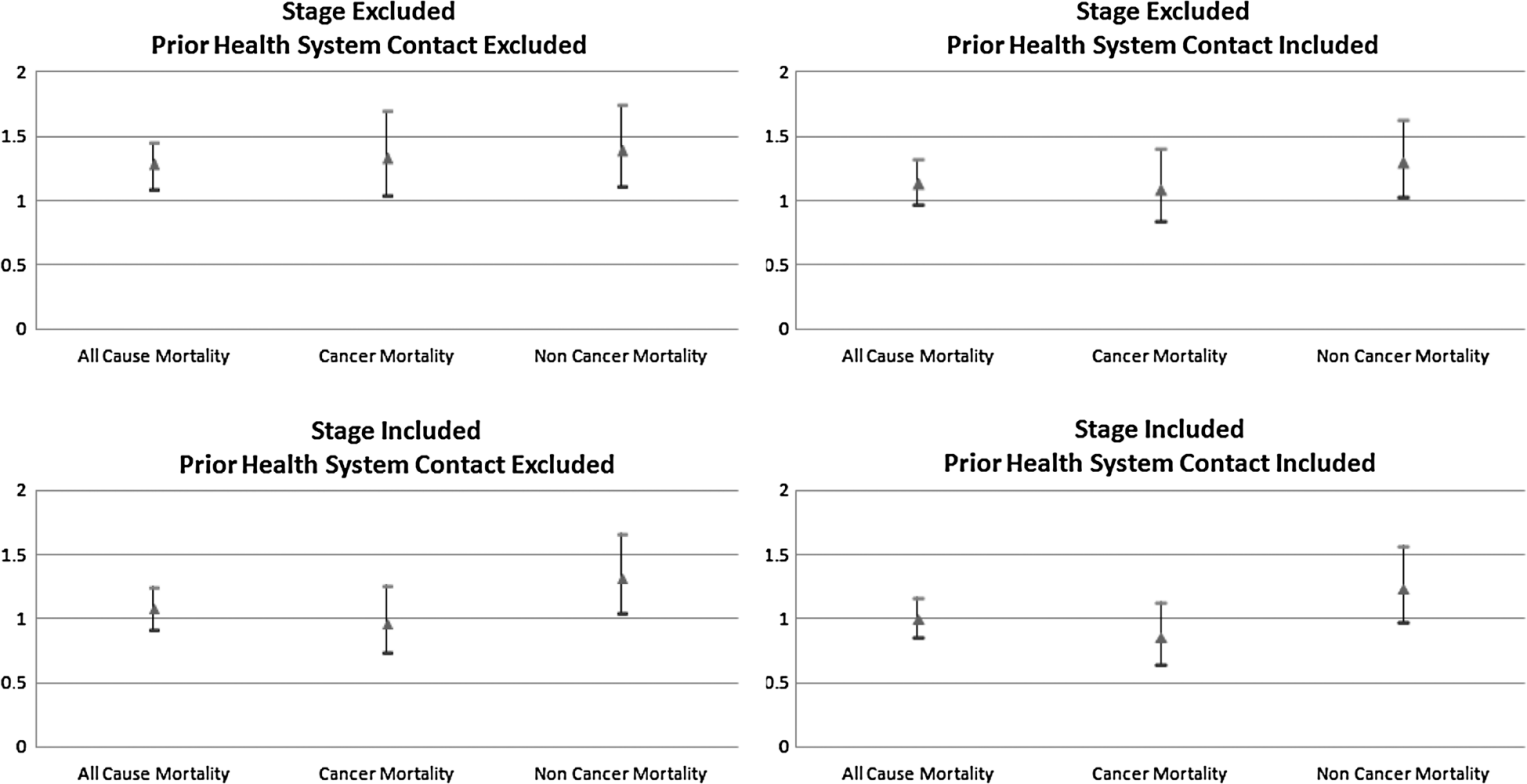
**Multivariate survival analyses: sensitivity analysis of adjusted hazard ratios for previously undiagnosed diabetes.** This figure presents the results of four sets of three multivariate survival analyses, which evaluate the sensitivity of the baseline findings reported in Table 3 (upper right quadrant in Figure) based on the inclusion and exclusion of cancer stage at diagnosis and measures of prior health system contact. Within each quadrant are the results of 3 separate multivariate analyses, one for each mortality outcome. Triangles represent the adjusted hazard ratios for previously undiagnosed diabetes (compared to previously diagnosed diabetes). Ticks represent the upper and lower bounds of the 95% confidence interval corresponding to the hazard ratio. Confidence intervals that do not overlap 1.0 are statistically significant at a threshold of P = 0.05.

Overall, 980/2,418 (40.5%) died during the observation period: 40.2% of those with previously diagnosed diabetes and 41.5% of those with previously undiagnosed diabetes. The estimated median survival based on Kaplan-Meier analysis was 68.6 months in those with previously diagnosed diabetes and 62.3 months in those with previously undiagnosed diabetes. In multivariate survival analysis that included all covariates in Table 1 except cancer stage and the two measures of prior health system contact (types of physician contact and preventive services), previously undiagnosed (compared to previously diagnosed) diabetes was associated with significantly higher all-cause (HR = 1.25; 95% CI 1.08 – 1.45; P < .01), cancer (HR = 1.33; 95% CI 1.04 – 1.70; P = 0.03), and other-cause mortality (HR = 1.39; 95% CI 1.11 – 1.75; P = 0.01). Adding measures of prior health system contact to the (base-case) model reduced the magnitude and statistical significance of the HR for previously undiagnosed diabetes on all three measures of mortality: all-cause (HR = 1.13; 95% CI 0.97 – 1.32; P = 0.11), cancer (HR = 1.08; 95% CI 0.84 – 1.40; P = 0.54), and other-cause mortality (HR = 1.29; 95% CI 1.02 – 1.63; P = 0.03). (Table 3) Adding cancer stage further attenuated the associations between previously undiagnosed diabetes and mortality. However, as discussed in the Methods, these models may be over-adjusted since cancer stage was hypothesized to be an intermediate variable between previously undiagnosed diabetes and mortality (Figure 2).

**Table 3:** Multivariate survival analysis

	**All-cause mortality**				**Cancer mortality**				**Other-cause mortality**			
	**HR**	**95% CI**		**P-Value**	**HR**	**95% CI**		**P-Value**	**HR**	**95% CI**		**P-Value**
		**Lower**	**Upper**			**Lower**	**Upper**			**Lower**	**Upper**	
**Diabetes status**												
**Previously diagnosed**	**Reference**											
**Previously undiagnosed**	1.13	0.97	1.32	0.11	1.08	0.84	1.40	0.54	1.29	1.02	1.63	0.03
**Age at cancer diagnosis (years)**												
**66-70**	**Reference**											
**71-75**	1.12	0.88	1.44	0.35	1.31	0.89	1.92	0.18	0.87	0.57	1.31	0.50
**76-80**	1.47	1.16	1.85	<0.01	1.37	0.93	2.02	0.11	1.40	0.96	2.02	0.08
**>80**	1.90	1.53	2.36	<0.0001	1.46	1.02	2.09	0.04	2.16	1.54	3.03	<0.0001
**Race/ethnicity**												
**White**	**Reference**											
**Black**	1.08	0.83	1.40	0.58	1.07	0.69	1.66	0.75	0.95	0.61	1.47	0.82
**Hispanic**	0.86	0.61	1.21	0.37	1.02	0.61	1.72	0.93	1.15	0.69	1.89	0.60
**Other**	0.92	0.56	1.51	0.74	0.77	0.28	2.08	0.60	1.07	0.53	2.17	0.86
**Year of diagnosis**												
**2001**	**Reference**											
**2002**	1.06	0.89	1.27	0.50	1.23	0.89	1.70	0.22	1.20	0.91	1.57	0.19
**2003**	1.07	0.89	1.29	0.50	1.05	0.75	1.47	0.79	1.09	0.81	1.45	0.58
**2004**	0.90	0.73	1.10	0.29	1.05	0.73	1.50	0.80	0.90	0.66	1.24	0.53
**2005**	1.02	0.83	1.25	0.86	1.15	0.81	1.63	0.44	0.95	0.68	1.33	0.75
**Estrogen (ER) and progesterone (PR) receptor status**												
**ER or PR positive**	**Reference**											
**ER and PR positive**	0.84	0.70	1.01	0.07	0.64	0.47	0.88	0.01	0.98	0.73	1.31	0.89
**ER and PR negative**	1.31	1.04	1.64	0.02	1.56	1.10	2.21	0.01	0.90	0.61	1.34	0.61
**Unknown/missing**	0.86	0.70	1.05	0.14	0.58	0.40	0.83	<0.01	1.06	0.77	1.45	0.74
**Histologic grade**												
**1**	**Reference**											
**2**	1.14	0.94	1.39	0.18	2.24	1.42	3.53	<0.01	0.86	0.66	1.12	0.27
**3**	1.54	1.26	1.88	<0.0001	3.17	2.01	5.01	<0.0001	1.20	0.90	1.59	0.21
**4**	1.15	0.76	1.72	0.51	3.32	1.65	6.68	<0.01	0.75	0.39	1.46	0.40
**Unknown/missing**	1.66	1.32	2.08	<0.0001	4.04	2.48	6.57	<0.0001	0.91	0.64	1.28	0.58
**NCI Comorbidity Index**												
**0**	**Reference**											
**1**	1.37	1.17	1.61	<0.01	0.99	0.76	1.30	0.96	1.64	1.27	2.12	<0.01
**≥ 2**	1.83	1.55	2.16	<0.0001	1.11	0.82	1.49	0.51	2.53	1.95	3.27	<0.0001
**Indicators of poor performance**												
**0**	**Reference**											
**≥ 1**	1.27	1.10	1.46	<0.01	1.00	0.78	1.28	0.97	1.66	1.33	2.08	<0.0001
**Types of physician visits**												
**None**	**Reference**											
**Primary care and medical specialist**	0.56	0.44	0.72	<0.0001	0.44	0.30	0.65	<0.0001	0.61	0.40	0.93	0.02
**Primary care, no medical specialist**	0.70	0.54	0.92	0.01	0.65	0.43	0.98	0.04	0.63	0.39	1.00	0.05
**Medical specialist, no primary care**	0.72	0.54	0.96	0.02	0.43	0.26	0.70	<0.01	1.11	0.70	1.75	0.66
**Other specialist only**	1.02	0.69	1.50	0.94	0.76	0.39	1.46	0.40	1.04	0.55	1.97	0.91
**Preventive services**												
**0**	**Reference**											
**1**	0.78	0.63	0.96	0.02	0.78	0.55	1.10	0.15	0.99	0.69	1.42	0.95
**≥ 2**	0.53	0.43	0.64	<0.0001	0.41	0.29	0.57	<0.0001	0.73	0.52	1.02	0.07
**Percent in census tract with some college**												
**<25%**	**Reference**											
**≥25%**	1.09	0.96	1.24	0.20	1.16	0.92	1.46	0.21	1.24	1.01	1.52	0.04
**Percent in census tract living in poverty**												
**<5%**	**Reference**											
**5-7%**	0.97	0.80	1.19	0.79	0.94	0.67	1.31	0.72	0.97	0.72	1.30	0.82
**8-12%**	0.94	0.79	1.12	0.48	0.80	0.59	1.10	0.17	0.92	0.71	1.20	0.55
**>12%**	1.07	0.90	1.27	0.45	0.93	0.69	1.25	0.61	0.93	0.71	1.22	0.62
**Type of geographic area**												
**Large metropolitan**	**Reference**											
**Metropolitan**	0.99	0.86	1.15	0.90	0.80	0.62	1.03	0.09	0.92	0.73	1.15	0.47
**Urban**	0.97	0.75	1.25	0.80	0.63	0.38	1.06	0.08	0.77	0.49	1.22	0.26
**Less urban/rural**	0.84	0.67	1.04	0.11	0.60	0.39	0.90	0.01	1.09	0.79	1.51	0.58

*HR*: Hazard ratio. *CI*: Confidence interval.

In the base-case survival analyses, (Table 3) other factors associated with a significantly higher cancer mortality rate were age > 80 years at cancer diagnosis, ER- and PR-negative disease, and higher grade histology. Primary care physician and/or medical specialist contact prior to cancer diagnosis, receipt of ≥ 2 preventive services, and ER- and PR-positive disease were associated with a significantly lower cancer mortality rate. Factors associated with significantly higher other-cause mortality were age > 80 years, NCI Comorbidity Index score of 1 or ≥ 2, at least one indicator of poor performance, and living in a census tract with ≥ 25% college educated. Primary care physician contact prior to cancer diagnosis was associated with significantly lower other-cause mortality.

## Discussion

Pre-existing diabetes is associated with increased risk of breast cancer [1] and adverse outcomes [2]. Biological and other factors underlying the association between pre-existing diabetes and breast cancer could differ in those whose diabetes remains undiagnosed until cancer. In this study, we identified a cohort of women diagnosed with both breast cancer *and diabetes*. We divided the cohort into two groups: those with previously diagnosed diabetes and those with previously undiagnosed diabetes. We then described risk factors and outcomes associated with previously undiagnosed compared to previously diagnosed diabetes.

More than one quarter of the patients had previously undiagnosed diabetes, which is somewhat lower than rates of previously undiagnosed diabetes based on glucose testing in the general population [27] or in those with breast cancer [5]. Ours may be a conservative estimate since we used medical claims from a six-month period around the diagnosis of breast cancer to identify previously undiagnosed diabetes, and the algorithm we used has a reported sensitivity of approximately 74% [25]. Among the risk factors for previously undiagnosed diabetes was low level of health system contact prior to cancer. Specifically, those with lower utilization of preventive services and less contact with primary care physicians or medical specialists were at significantly higher risk of previously undiagnosed diabetes. We did not include cancer stage as a covariate in the multivariate analyses of factors associated with previously undiagnosed diabetes, because our causal pathway diagram indicates it is a collider [47-49] in this instance. Consequently, conditioning on stage could have opened a biasing pathway (the analysis may have identified an association where none exists) between the vector of patient characteristics and previously undiagnosed diabetes.

Our findings show that previously undiagnosed diabetes is associated with higher odds of being diagnosed with advanced stage breast cancer. Possible explanations include exacerbated biological mechanisms related to hyperglycemia, hyperinsulinemia, and inflammation in undiagnosed diabetes, which can result in tumor cell proliferation and metastases [16-18]. However, since we did not have information on insulin and glucose levels, or on duration of previously undiagnosed diabetes, these findings should be considered as hypothesis-generating, requiring laboratory data and information on unobserved confounders for further evaluation. Also, although in the causal diagram previously undiagnosed diabetes precedes advanced-stage cancer diagnosis, since previously undiagnosed diabetes status was ascertained at the same time as cancer stage, we cannot conclude that previously undiagnosed diabetes caused advanced stage cancer in our study.

The potential for confounding in this analysis due to shared risk factors was significant, as illustrated in the causal pathway diagram we developed. Since previous research shows that limited health system contact is associated with advanced stage cancer at diagnosis [21-23], and limited health system contact was associated with previously undiagnosed diabetes in this study, we sought to block this “biasing pathway” [47] by including measures of prior health system contact in the final multivariate model of stage, and in doing so confirmed the earlier findings [21-23]. Also, we sought to minimize other sources of potential confounding – due to unobserved factors that place individuals at higher risk of diabetes -- by limiting the cohort to those with diabetes. Unmeasured potential confounders include diabetes severity measures, body-mass index, diabetes treatment/medications, and other health behavior.

Finally, previously undiagnosed diabetes was associated with significantly increased mortality, but this effect was limited to death from causes other than cancer. This suggests patients with undiagnosed diabetes are sicker overall, and are more likely to die from “competing risks” rather than directly from breast cancer. Any effect of previously undiagnosed diabetes on cancer mortality appears to be mediated entirely by advanced stage as an intermediate risk factor, and poor prior health system contact as a confounding factor. It is unclear whether these findings specific to breast cancer can be generalized to other types of cancers, where the impact of diabetes on cancer treatment and outcomes may differ. As with the analyses of previously undiagnosed diabetes and stage, there are unmeasured potential confounders in the survival analyses, including cancer treatment, surveillance, and diabetes-related complications.

Our study has several limitations. As discussed above, the claims-based algorithm we used to identify diabetes has a validated sensitivity of 74.4% and specificity of 97.5% using a 2-year look-back period [25]. Therefore, we have likely missed cases of diabetes that would, for instance, have been identified through electronic medical records containing detailed laboratory and oral medications data. Also, we have described diabetes first detected three months before to three months after cancer as previously undiagnosed diabetes, which implies that it was present but undetected prior to that. However, simply by chance, it is likely that some patients had new onset diabetes during this period. Further, it is possible that some of the diabetes cases we identified as previously undiagnosed would have been reclassified as previously diagnosed had we extended the look-back period of the algorithm from 24 to 36 months. However, this would have resulted in excluding all patients aged 67, who would not have had at least 36 months of Medicare eligibility prior to the diagnosis of cancer.

This study was conducted prior to the implementation of the Medicare Modernization Act (MMA), which introduced new coverage for diabetes and other screening services in 2005 [50]. Introduction of these services is designed to improve early detection of diabetes and other important conditions. Therefore, rates of previously undiagnosed diabetes could change as a result of MMA. In addition to affecting the incidence of previously undiagnosed diabetes, MMA could impact the services included in the preventive services measure of prior health system contact. Since some of the new services directly impact diabetes, it is possible that associations between level of preventive services use and previously undiagnosed diabetes would become stronger as a result, but that the overall incidence of previously undiagnosed diabetes has declined through improved coverage of preventive services.

We considered propensity score analysis. However, elucidating our pathway diagram required that we examine the effects of including/excluding specific covariables, e.g. stage and measures of prior health system contact, on the associations between previously undiagnosed diabetes and outcomes, which would not have been possible had we summarized these effects in a single propensity score. Finally, it was not our intent in this study to compare the outcomes of those with versus without diabetes. Therefore, we did not include a control group of patients who did not have diabetes.

## Conclusions

When compared to previously diagnosed diabetes, previously undiagnosed diabetes is associated with increased risk of advanced stage cancer. While previously undiagnosed diabetes does not appear to confer additional risk of cancer mortality, it is associated with increased risk of death due to other causes. The fact that limited prior health system contact was a risk factor for previously undiagnosed diabetes, advanced stage cancer, and mortality (cancer and other cause) underscores the importance of continuing to strengthen Medicare coverage for, and promote use of, routine primary care physician visits and preventive services, especially for those with shared risk factors for both breast cancer and diabetes.

## Competing interests

The authors declare that they have no competing interests.

## Authors’ contributions

RIG, MDD and MLG acquired the data and developed the initial analysis plan. JMV reviewed and provided comments on the analysis plan. RIG wrote the initial version of the manuscript. RIG and MLG created all tables and figures. All authors contributed to the revisions of the manuscript.

## Pre-publication history

The pre-publication history for this paper can be accessed here:

http://www.biomedcentral.com/1471-2407/12/613/prepub
